# Supplementary material for: The Known, the Unknown and the Future of the Pathophysiology of Endometriosis
Source: Int J Mol Sci. 2024 May 27;25(11):5815. doi: 10.3390/ijms25115815 (PMC11172035; doi:10.3390/ijms25115815)
Supplement: Supplementary file 1 [file ijms-25-05815-s001.zip › ijms-2971196-supplementary.pdf]

**Supplementary Table S1: Baboon model to study the pathogenesis of Endometriosis**

| **References** | **Main Focus of the study** | **Year** |
|---|---|---|
| DaRif et al.[1] | Case of bacterial peritonitis in a confirmed case of endometriosis | 1984 |
| D′ Hooghe et al.[2] | Evolution of spontaneous endometriosis | **1992** |
| Cornillie et al.[3] | Morphological characteristics of spontaneous endometriosis | **1992** |
| Shalev et al.[4] | Case of a stromal tumor in a case of endometriosis | **1992** |
| D′ Hooghe et al.[5] | Development of a model of retrograde menstruation | **1994** |
| D′ Hooghe et al.[6] | Cycle fecundity of proven fertility with minimal endometriosis | **1994** |
| Braundmeier et al.[7] | Findings and theories on the development of endometriosis and disease progression and the effectiveness of therapeutic targets and implication | **2009** |
| D′ Hooghe et al.[8] | Increased prevalence and recurrence of retrograde menstruation in spontaneous endometriosis | **1996** |
| D′ Hooghe et al.[9] | The effects of immunosuppression on development and progression of endometriosis | **1995** |
| D′ Hooghe et al.[10] | Intrapelvic injection of menstrual endometrium causes endometriosis | **1995** |
| D′ Hooghe et al.[11] | Peritoneal fluid volume and steroid hormone concentration with and without endometriosis | **1995** |
| D′ Hooghe et al.[12] | A serial section study of visually normal peritoneum with and without spontaneous endometriosis | **1995** |
| D′ Hooghe et al.[13] | Anti-endometrial lymphocytotoxicity and natural killer cell activity in endometriosis | **1995** |
| D′ Hooghe et al.[14] | Effect of endometriosis on white blood cell and subpopulations in peripheral blood and peritoneal fluid | **1996** |
| D′ Hooghe et al.[15] | Development of spontaneous endometriosis | **1996** |
| D′ Hooghe et al.[16] | Disease progression during serial laparoscopies over 30 months | **1996** |
| D′ Hooghe et al.[17] | The cycle pregnancy rate during different stages of endometriosis | **1996** |
| D′ Hooghe et al.[18] | Luteinized unruptured follicle syndrome during endometriosis | **1996** |
| D′ Hooghe et al.[19] | Clinical relevance of the baboon as a model for endometriosis | **1997** |
| D′ Hooghe et al.[20] | The effect of pregnancy on endometriosis | **1997** |
| D′ Hooghe et al.[21] | Pelvic inflammation induced by diagnostic laparoscopy | **1999** |
| Strakova et al.[22] | Interleukin -1β induces the expression of IGFBP-1 during decidualization | **2000** |
| Strakova et al. [23] | Inhibition of matrix metalloproteinases and prevention of synthesis of IGFBP-1 during decidualization | 2003 |
| Cameo et al.[24] | Decidualization regulates the expression of the endometrial chorionic gonadotropin receptor | 2006 |
| Ochoa-Bernal et al.[25] | Physiologic Events of Embryo Implantation and Decidualization | 2020 |
| D′ Hooghe et al.[26] | Effect of menstruation and intrapelvic injection of endometrium on inflammatory parameters of peritoneal fluid | 2001 |
| Fazleabas et al.[27] | A modified baboon model for endometriosi | **2002** |
| Fazleabas et al.[28] | Endometriosis in the baboon | 2004 |
| Fazleabas[29] | A baboon model for inducing endometriosis | 2006 |
| Kyama et al. [30] | Baboon model for the study of endometriosis | 2007 |
| D′ Hooghe et al[31] | Non human primate models for translational research in | 2009 |

| | | |
|---|---|---|
| | endometriosis | |
| Nair et al.[32] | An efficient model of human endometriosis by induced unopposed estrogenicity in baboons | 2016 |
| Fazleabas et al.[33] | Endometrial function: cell specific changes in the uterine environment | **2002** |
| D′ Hooghe et al.[34] | Endometriosis, retrograde menstruation and peritoneal inflammation | **2002** |
| D′ Hooghe et al.[35] | Endometriosis and subfertility | **2003** |
| Hastings et al.[36] | Implications for fertility | 2006 |
| Fazleabas at al.[37] | Steroid receptor and aromatase expression in endometriosis | **2003** |
| Moore et al.[38] | Cases of spontaneous ovarian tumors in baboons | **2003** |
| Barrier et al.[39] | Adenomyosis in the baboon is associated with primary infertility | **2004** |
| Barrier et al.[40] | Efficacy of anti-tumor necrosis factor therapy in the treatment of spontaneous endometriosis | **2004** |
| Falconer et al. [41] | The effect of endometriosis, cycle stage, lymphocyte suppression and pregnancy on CA-125 levels in peritoneal fluid and serum | **2005** |
| D′ Hooghe et al.[42] | Fundamental and preclinical research | **2004** |
| Gashaw et al.[43] | Induced endometriosis in the baboon (Papio anubis) increases the expression of the proangiogenic factor CYR61 (CCN1) in eutopic and ectopic endometria. | **2006** |
| Hastings et al.[44] | The alteration of the estrogen early response gene FOS | **2006** |
| Falconer et al. [45] | Efficacy of anti-TNF monoclonal antibody in reducing established endometriosis | **2006** |
| Jones et al. [46] | Morphological and glycosylation changes associated with the endometrium and ectopic lesions | **2006** |
| D′ Hooghe et al.[47] | Recombinant human TNFRSF1A (r-hTBP1) inhibits the development of endometriosis | **2006** |
| Kyama et al.[48] | Effect of recombinant human TNF-binding protein-1 and GnRH antagonist | **2006** |
| Afshar et al.[49] | The role of chorionic gonadotropin and Notch1 in implantation | **2007** |
| Afshar et al.[50] | Notch1 regulation by chorionic gonadotropin and progesterone in endometrial stromal cells and modulates decidualization | 2012 |
| Su et al. [51] | Decreased Notch pathway signaling in the endometrium | 2015 |
| Song et al.[52] | Interleukin-6 (IL-6) Activates the NOTCH1 Signaling Pathway Through E-Proteins in Endometriotic Lesions | 2020 |
| Barrier et al.[53] | Endometriosis involving the ileocaecal junction with regional lymph node involvement | **2007** |
| Kim et al.[54] | Altered expression of HOXA10 in endometriosis: potential role in decidualization. | **2007** |
| Lebovic et al.[55] | PPAR-gamma receptor ligand induces regression of endometrial explants | **2007** |
| Falconer et al.[56] | Effects of anti-TNF-mAb treatment on pregnancy in baboons with induced endometriosis | **2008** |
| Bennet et al.[57] | Endometrial and cervical polyps | **2009** |
| Kyama et al.[58] | Role of cytokines in the endometrial-peritoneal cross-talk and development of endometriosis. | **2009** |
| Jones et al. [59] | Ultrastructure of ectopic peritoneal lesions | **2009** |

| | | |
|---|---|---|
| Winterhager et al.[60] | Connexin expression pattern in the endometrium of baboons is influenced by hormonal changes and the presence of endometriotic lesions | **2009** |
| Banerjee et al.[61] | Endometrial responses to embryonic signals | **2010** |
| Fazleabas[62] | Progesterone resistance in endometriosis | **2010** |
| Joshi et al. [63] | Progesterone resistance in endometriosis Is modulated by the Altered Expression of MicroRNA-29c and FKBP4. | 2017 |
| Sherwin et al.[64] | Endometrial response to chorionic gonadotropin | **2010** |
| Hapangama et al.[65] | Expression of regulators of cell-fate | **2010** |
| Ilad et al.[66] | Immunohistochemical study of the ubiquitin-nuclear factor-kB pathway | **2010** |
| Braundmeier et al.[67] | Extracellular matrix metalloproteinase inducer expression | **2010** |
| Brosens et al.[68] | Proteomic analysis of endometrium | **2010** |
| Lebovic et al.[69] | Peroxisome proliferator-activated receptor-(gamma) receptor ligand | **2010** |
| Hey-Cunningham et al.[70] | Endometrial stromal cells and immune cell populations within lymph nodes | **2011** |
| Morris et al.[71] | Cofilin and slingshot localization in the epithelium of uterine endometrium changes during the menstrual cycle and in endometriosis | **2011** |
| Kemnitz et al.[72] | Calorie restriction and aging in nonhuman primates | **2011** |
| Harirchian et al.[73] | Lesion kinetics in a non-human primate model of endometriosis | **2012** |
| Campo et al.[74] | Adenomyosis and infertility | **2012** |
| Afshar et al.[75] | Changes in eutopic endometrial gene expression during the progression of experimental endometriosis | **2013** |
| Langoi et al.[76] | Aromatase inhibitor treatment limits progression of peritoneal endometriosis | **2013** |
| Jagirdar et al.[77] | Pleuro-pulmonary in endometriosis | **2013** |
| Donnez et al.[78] | Induction of endometriotic nodules in an experimental baboon model mimicking human deep nodular lesions. | **2013** |
| Donnez et al.[79] | Nerve fiber density in deep nodular endometriotic lesions | **2013** |
| Orellana et al.[80] | Role of collective cell migration and nerve fiber density in the development of deep nodular endometriosis | 2017 |
| Kyama et al.[81] | Menstrual endometrial supernatant may induce stromal endometriosis | **2014** |
| Sugihara et al.[82] | Development of pro-apoptotic peptides as potential therapy for peritoneal endometriosis | **2014** |
| Fazleabas at al.[83] | Endometriosis-induced changes in regulatory T cells | **2015** |
| Braundmeier et al.[84] | Induction of endometriosis alters the peripheral and endometrial regulatory T cell population | 2012 |
| Joshi et al.[85] | Altered expression of microRNA-451 in eutopic endometrium | **2015** |
| Nothnick et al. [86] | Serum miR-451a Levels Are Significantly Elevated in Endometriosis | 2017 |
| Kim et al.[87] | Aberrant activation of signal transducer and activator of transcription-3 (STAT3) signaling in endometriosis | **2015** |
| Yoo et al.[88] | Protein Inhibitor of Activated STAT3 (PIAS3) Is Down-Regulated in Eutopic Endometrium | 2016 |
| Su et al. [89] | Implantation and Establishment of Pregnancy | **2015** |

| | | |
|---|---|---|
| Baumann et al. [90] | Arginine methyltransferases mediate an epigenetic ovarian response to endometriosis. | **2015** |
| Zhang et al.[91] | Cellular Changes Consistent With Epithelial-Mesenchymal Transition and Fibroblast-to-Myofibroblast Transdifferentiation in the Progression of Experimental Endometriosis i | **2016** |
| Parkin et al.[92] | Uterine Leukocyte Function and Dysfunction: A Hypothesis on the Impact of Endometriosis. | **2016** |
| Slayden[93] | Translational In Vivo Models for Women's Health: The Nonhuman Primate Endometrium--A Predictive Model for Assessing Steroid Receptor Modulators. | **2016** |
| Hussein et al.[94] | c-Jun NH2-terminal kinase inhibitor bentamapimod reduces induced endometriosis | **2016** |
| Taylor et al.[95] | Effect of simvastatin in endometriosis | **2017** |
| Cosar et al.[96] | Serum MicroRNA Biomarkers Regulated by Simvastatin in Endometriosis. | 2019 |
| Yoo et at.[97] | KRAS Activation and over-expression of SIRT1/BCL6 Contributes to the Pathogenesis of Endometriosis and Progesterone Resistance | 2017 |
| Stouffer et al.[98] | A Vital Model for Basic and Applied Research on Female Reproduction, Prenatal Development, and Women's Health | 2017 |
| Drury et al. [99] | The dynamic changes in the number of uterine natural killer cells are specific to the eutopic but not to the ectopic endometrium | **2018** |
| Chang et al.[100] | Overexpression of Four Joint Box-1 Protein (FJX1) in Eutopic Endometrium | **2018** |
| Hufnagel et al. [101] | Icon immunoconjugate treatment results in regression of red lesions | **2018** |
| Nothnick et al.[102] | Macrophage Migration Inhibitory Factor Receptor, CD74, is Overexpressed in) Endometriotic Lesions and Modulates Endometriotic Epithelial Cell Survival and Interleukin 8 Expression | **2018** |
| Kim et al.[103] | Loss of HDAC3 results in nonreceptive endometrium and female infertility | **2019** |
| Hapangama et al.[104] | Endometriosis associated with abnormally located endometrial basalis-like (SSEA1+/SOX9+ | **2019** |
| Kirejczyk et al.[105] | Urogenital lesions | 2021 |
| Le et al.[106] | Effects of endometriosis on immunity and mucosal microbial community dynamics | 2022 |
| Poirier et al.[107] | An irreversible inhibitor of 17β-hydroxysteroid dehydrogenase type 1 inhibits estradiol synthesis in human endometriosis lesions and induces regression of the non-human primate endometriosis. | 2022 |

## References


1. DaRif, C.A.; Parker, R.F.; Schoeb, T.R. Endometriosis with bacterial peritonitis in a baboon. *Lab. Anim. Sci.* **1984**, *34*, 491–493.
2. D'Hooghe, T.M.; Bambra, C.S.; Isahakia, M.; Koninckx, P.R. Evolution of spontaneous endometriosis in the baboon (*Papio anubis*, *Papio cynocephalus*) over a 12-month period. *Fertil. Steril.* **1992**, *58*, 409–412.
3. Cornillie, F.J.; D'Hooghe, T.M.; Bambra, C.S.; Lauweryns, J.M.; Isahakia, M.; Koninckx, P.R. Morphological characteristics of spontaneous endometriosis in the baboon (*Papio anubis* and *Papio cynocephalus*). *Gynecol. Obstet. Investig.* **1992**, *34*, 225–228. https://doi.org/10.1159/000292766.
4. Shalev, M.; Ciurea, D.; Deligdisch, L. Endometriosis and stromal tumor in a baboon (*Papio hamadryas*). *Lab. Anim. Sci.* **1992**, *42*, 204–208.

5. D'Hooghe, T.M.; Bambra, C.S.; Suleman, M.A.; Dunselman, G.A.; Evers, H.L.; Koninckx, P.R. Development of a model of retrograde menstruation in baboons (*Papio anubis*). *Fertil. Steril.* **1994**, *62*, 635–638.
6. D'Hooghe, T.M.; Bambra, C.S.; Koninckx, P.R. Cycle fecundity in baboons of proven fertility with minimal endometriosis. *Gynecol. Obstet. Investig.* **1994**, *37*, 63–65. https://doi.org/10.1159/000292524.
7. Braundmeier, A.G.; Fazleabas, A.T. The non-human primate model of endometriosis: Research and implications for fecundity. *Mol. Hum. Reprod.* **2009**, *15*, 577–586. https://doi.org/10.1093/molehr/gap057.
8. D'Hooghe, T.M.; Bambra, C.S.; Raeymaekers, B.M.; Koninckx, P.R. Increased prevalence and recurrence of retrograde menstruation in baboons with spontaneous endometriosis. *Hum. Reprod.* **1996**, *11*, 2022–2025. https://doi.org/10.1093/oxfordjournals.humrep.a019537.
9. D'Hooghe, T.M.; Bambra, C.S.; Raeymaekers, B.M.; De Jonge, I.; Hill, J.A.; Koninckx, P.R. The effects of immunosuppression on development and progression of endometriosis in baboons (*Papio anubis*). *Fertil. Steril.* **1995**, *64*, 172–178.
10. D'Hooghe, T.M.; Bambra, C.S.; Raeymaekers, B.M.; De Jonge, I.; Lauweryns, J.M.; Koninckx, P.R. Intrapelvic injection of menstrual endometrium causes endometriosis in baboons (*Papio cynocephalus* and *Papio anubis*). *Am. J. Obstet. Gynecol.* **1995**, *173*, 125–134. https://doi.org/10.1016/0002-9378(95)90180-9.
11. D'Hooghe, T.M.; Bambra, C.S.; Kazungu, J.; Koninckx, P.R. Peritoneal fluid volume and steroid hormone concentrations in baboons with and without either spontaneous minimal/mild endometriosis or the luteinized unruptured follicle syndrome. *Arch. Gynecol. Obstet.* **1995**, *256*, 17–22. https://doi.org/10.1007/bf00634343.
12. D'Hooghe, T.M.; Bambra, C.S.; De Jonge, I.; Machai, P.N.; Korir, R.; Koninckx, P.R. A serial section study of visually normal posterior pelvic peritoneum from baboons (*Papio cynocephalus*, *Papio anubis*) with and without spontaneous minimal endometriosis. *Fertil. Steril.* **1995**, *63*, 1322–1325. https://doi.org/10.1016/s0015-0282(16)57618-3.
13. D'Hooghe, T.M.; Scheerlinck, J.P.; Koninckx, P.R.; Hill, J.A.; Bambra, C.S. Anti-endometrial lymphocytotoxicity and natural killer cell activity in baboons (*Papio anubis* and *Papio cynocephalus*) with endometriosis. *Hum. Reprod.* **1995**, *10*, 558–562. https://doi.org/10.1093/oxfordjournals.humrep.a135988.
14. D'Hooghe, T.M.; Hill, J.A.; Oosterlynck, D.J.; Koninckx, P.R.; Bambra, C.S. Effect of endometriosis on white blood cell subpopulations in peripheral blood and peritoneal fluid of baboons. *Hum. Reprod.* **1996**, *11*, 1736–1740. https://doi.org/10.1093/oxfordjournals.humrep.a019478.
15. D'Hooghe, T.M.; Bambra, C.S.; Raeymaekers, B.M.; Koninckx, P.R. Development of spontaneous endometriosis in baboons. *Obstet. Gynecol.* **1996**, *88*, 462–466. https://doi.org/10.1016/0029-7844(96)00205-0.
16. D'Hooghe, T.M.; Bambra, C.S.; Raeymaekers, B.M.; Koninckx, P.R. Serial laparoscopies over 30 months show that endometriosis in captive baboons (*Papio anubis*, *Papio cynocephalus*) is a progressive disease. *Fertil. Steril.* **1996**, *65*, 645–649.
17. D'Hooghe, T.M.; Bambra, C.S.; Raeymaekers, B.M.; Riday, A.M.; Suleman, M.A.; Koninckx, P.R. The cycle pregnancy rate is normal in baboons with stage I endometriosis but decreased in primates with stage II and stage III-IV disease. *Fertil. Steril.* **1996**, *66*, 809–813.
18. D'Hooghe, T.M.; Bambra, C.S.; Raeymaekers, B.M.; Koninckx, P.R. Increased incidence and recurrence of recent corpus luteum without ovulation stigma (luteinized unruptured follicle syndrome?) in baboons with endometriosis. *J. Soc. Gynecol. Investig.* **1996**, *3*, 140–144.
19. D'Hooghe, T.M. Clinical relevance of the baboon as a model for the study of endometriosis. *Fertil. Steril.* **1997**, *68*, 613–625. https://doi.org/10.1016/S0015-0282(97)00277-X.
20. D'Hooghe, T.M.; Bambra, C.S.; De Jonge, I.; Lauweryns, J.M.; Raeymaekers, B.M.; Koninckx, P.R. The effect of pregnancy on endometriosis in baboons (*Papio anubis*, *Papio cynocephalus*). *Arch. Gynecol. Obstet.* **1997**, *261*, 15–19. https://doi.org/10.1007/s004040050191.
21. D'Hooghe, T.M.; Bambra, C.S.; Raeymaekers, B.M.; Hill, J.A. Pelvic inflammation induced by diagnostic laparoscopy in baboons. *Fertil. Steril.* **1999**, *72*, 1134–1141. https://doi.org/10.1016/s0015-0282(99)00406-9.
22. Strakova, Z.; Srisuparp, S.; Fazleabas, A.T. Interleukin-1beta induces the expression of insulin-like growth factor binding protein-1 during decidualization in the primate. *Endocrinology* **2000**, *141*, 4664–4670. https://doi.org/10.1210/endo.141.12.7810.
23. Strakova, Z.; Szmidt, M.; Srisuparp, S.; Fazleabas, A.T. Inhibition of matrix metalloproteinases prevents the synthesis of insulin-like growth factor binding protein-1 during decidualization in the baboon. *Endocrinology* **2003**, *144*, 5339–5346. https://doi.org/10.1210/en.2003-0471.
24. Cameo, P.; Szmidt, M.; Strakova, Z.; Mavrogianis, P.; Sharpe-Timms, K.L.; Fazleabas, A.T. Decidualization regulates the expression of the endometrial chorionic gonadotropin receptor in the primate. *Biol. Reprod.* **2006**, *75*, 681–689. https://doi.org/10.1095/biolreprod.106.051805.

25. Ochoa-Bernal, M.A.; Fazleabas, A.T. Physiologic Events of Embryo Implantation and Decidualization in Human and Non-Human Primates. *Int. J. Mol. Sci.* **2020**, *21*, 1973. https://doi.org/10.3390/ijms21061973.
26. D'Hooghe, T.M.; Bambra, C.S.; Xiao, L.; Peixe, K.; Hill, J.A. Effect of menstruation and intrapelvic injection of endometrium on inflammatory parameters of peritoneal fluid in the baboon (*Papio anubis* and *Papio cynocephalus*). *Am. J. Obstet. Gynecol.* **2001**, *184*, 917–925. https://doi.org/10.1067/mob.2001.111715.
27. Fazleabas, A.T.; Brudney, A.; Gurates, B.; Chai, D.; Bulun, S. A modified baboon model for endometriosis. *Ann. N. Y. Acad. Sci.* **2002**, *955*, 308–317; discussion 340-302, 396-406. https://doi.org/10.1111/j.1749-6632.2002.tb02791.x.
28. Fazleabas, A.T.; Brudney, A.; Chai, D.; Mwenda, J. Endometriosis in the baboon. *Gynecol. Obstet. Investig.* **2004**, *57*, 46–47.
29. Fazleabas, A.T. A baboon model for inducing endometriosis. *Methods Mol. Med.* **2006**, *121*, 95–99. https://doi.org/10.1385/1-59259-983-4:093.
30. Kyama, C.M.; Mihalyi, A.; Chai, D.; Simsa, P.; Mwenda, J.M.; D'Hooghe, T.M. Baboon model for the study of endometriosis. *Womens Health* **2007**, *3*, 637–646. https://doi.org/10.2217/17455057.3.5.637.
31. D'Hooghe, T.M.; Kyama, C.M.; Chai, D.; Fassbender, A.; Vodolazkaia, A.; Bokor, A.; Mwenda, J.M. Nonhuman primate models for translational research in endometriosis. *Reprod. Sci.* **2009**, *16*, 152–161. https://doi.org/10.1177/1933719108322430.
32. Nair, H.B.; Baker, R.; Owston, M.A.; Escalona, R.; Dick, E.J.; VandeBerg, J.L.; Nickisch, K.J. An efficient model of human endometriosis by induced unopposed estrogenicity in baboons. *Oncotarget* **2016**, *7*, 10857–10869. https://doi.org/10.18632/oncotarget.7516.
33. Fazleabas, A.T.; Strakova, Z. Endometrial function: Cell specific changes in the uterine environment. *Mol. Cell. Endocrinol.* **2002**, *186*, 143–147.
34. D'Hooghe, T.M.; Debrock, S. Endometriosis, retrograde menstruation and peritoneal inflammation in women and in baboons. *Hum. Reprod. Update* **2002**, *8*, 84–88. https://doi.org/10.1093/humupd/8.1.84.
35. D'Hooghe, T.M.; Debrock, S.; Hill, J.A.; Meuleman, C. Endometriosis and subfertility: Is the relationship resolved? *Semin. Reprod. Med.* **2003**, *21*, 243–254. https://doi.org/10.1055/s-2003-41330.
36. Hastings, J.M.; Fazleabas, A.T. A baboon model for endometriosis: Implications for fertility. *Reprod. Biol. Endocrinol.* **2006**, *4* (Suppl. 1), S7. https://doi.org/10.1186/1477-7827-4-S1-S7.
37. Fazleabas, A.T.; Brudney, A.; Chai, D.; Langoi, D.; Bulun, S.E. Steroid receptor and aromatase expression in baboon endometriotic lesions. *Fertil. Steril.* **2003**, *80* (Suppl. 2), 820–827. https://doi.org/10.1016/s0015-0282(03)00982-8.
38. Moore, C.M.; Hubbard, G.B.; Leland, M.M.; Dunn, B.G.; Best, R.G. Spontaneous ovarian tumors in twelve baboons: A review of ovarian neoplasms in non-human primates. *J. Med. Primatol.* **2003**, *32*, 48–56. https://doi.org/10.1034/j.1600-0684.2003.00002.x.
39. Barrier, B.F.; Malinowski, M.J.; Dick, E.J., Jr.; Hubbard, G.B.; Bates, G.W. Adenomyosis in the baboon is associated with primary infertility. *Fertil. Steril.* **2004**, *82* (Suppl. 3), 1091–1094. https://doi.org/10.1016/j.fertnstert.2003.11.065.
40. Barrier, B.F.; Bates, G.W.; Leland, M.M.; Leach, D.A.; Robinson, R.D.; Propst, A.M. Efficacy of anti-tumor necrosis factor therapy in the treatment of spontaneous endometriosis in baboons. *Fertil. Steril.* **2004**, *81* (Suppl. 1), 775–779. https://doi.org/10.1016/j.fertnstert.2003.09.034.
41. Falconer, H.; Bambra, C.S.; Chai, D.; Cornillie, F.J.; Hill, J.A.; D'Hooghe, T.M. The effect of endometriosis, cycle stage, lymphocyte suppression and pregnancy on CA-125 levels in peritoneal fluid and serum in baboons. *Hum. Reprod.* **2005**, *20*, 3033–3038. https://doi.org/10.1093/humrep/dei181.
42. D'Hooghe, T.M.; Debrock, S.; Kyama, C.M.; Chai, D.C.; Cuneo, S.; Hill, J.A.; Mwenda, J.M. Baboon model for fundamental and preclinical research in endometriosis. *Gynecol. Obstet. Investig.* **2004**, *57*, 43–46.
43. Gashaw, I.; Hastings, J.M.; Jackson, K.S.; Winterhager, E.; Fazleabas, A.T. Induced endometriosis in the baboon (*Papio anubis*) increases the expression of the proangiogenic factor CYR61 (CCN1) in eutopic and ectopic endometria. *Biol. Reprod.* **2006**, *74*, 1060–1066. https://doi.org/10.1095/biolreprod.105.049320.
44. Hastings, J.M.; Jackson, K.S.; Mavrogianis, P.A.; Fazleabas, A.T. The estrogen early response gene FOS is altered in a baboon model of endometriosis. *Biol. Reprod.* **2006**, *75*, 176–182. https://doi.org/10.1095/biolreprod.106.052852.
45. Falconer, H.; Mwenda, J.M.; Chai, D.C.; Wagner, C.; Song, X.Y.; Mihalyi, A.; Simsa, P.; Kyama, C.; Cornillie, F.J.; Bergqvist, A.; et al. Treatment with anti-TNF monoclonal antibody (c5N) reduces the extent of induced endometriosis in the baboon. *Hum. Reprod.* **2006**, *21*, 1856–1862. https://doi.org/10.1093/humrep/del044.

46. Jones, C.J.; Denton, J.; Fazleabas, A.T. Morphological and glycosylation changes associated with the endometrium and ectopic lesions in a baboon model of endometriosis. *Hum. Reprod.* **2006**, *21*, 3068–3080. https://doi.org/10.1093/humrep/del310.
47. D'Hooghe, T.M.; Nugent, N.P.; Cuneo, S.; Chai, D.C.; Deer, F.; Debrock, S.; Kyama, C.M.; Mihalyi, A.; Mwenda, J.M. Recombinant human TNFRSF1A (r-hTBP1) inhibits the development of endometriosis in baboons: A prospective, randomized, placebo- and drug-controlled study. *Biol. Reprod.* **2006**, *74*, 131–136. https://doi.org/10.1095/biolreprod.105.043349.
48. Kyama, C.M.; Overbergh, L.; Mihalyi, A.; Cuneo, S.; Chai, D.; Debrock, S.; Mwenda, J.M.; Mathieu, C.; Nugent, N.P.; D'Hooghe, T.M. Effect of recombinant human TNF-binding protein-1 and GnRH antagonist on mRNA expression of inflammatory cytokines and adhesion and growth factors in endometrium and endometriosis tissues in baboons. *Fertil. Steril.* **2008**, *89*, 1306–1313. https://doi.org/10.1016/j.fertnstert.2006.11.205.
49. Afshar, Y.; Stanculescu, A.; Miele, L.; Fazleabas, A.T. The role of chorionic gonadotropin and Notch1 in implantation. *J. Assist. Reprod. Genet.* **2007**, *24*, 296–302. https://doi.org/10.1007/s10815-007-9149-2.
50. Afshar, Y.; Miele, L.; Fazleabas, A.T. Notch1 is regulated by chorionic gonadotropin and progesterone in endometrial stromal cells and modulates decidualization in primates. *Endocrinology* **2012**, *153*, 2884–2896. https://doi.org/10.1210/en.2011-2122.
51. Su, R.W.; Strug, M.R.; Joshi, N.R.; Jeong, J.W.; Miele, L.; Lessey, B.A.; Young, S.L.; Fazleabas, A.T. Decreased Notch pathway signaling in the endometrium of women with endometriosis impairs decidualization. *J. Clin. Endocrinol. Metab.* **2015**, *100*, E433–E442. https://doi.org/10.1210/jc.2014-3720.
52. Song, Y.; Su, R.W.; Joshi, N.R.; Kim, T.H.; Lessey, B.A.; Jeong, J.W.; Fazleabas, A.T. Interleukin-6 (IL-6) Activates the NOTCH1 Signaling Pathway Through E-Proteins in Endometriotic Lesions. *J. Clin. Endocrinol. Metab.* **2020**, *105*, 1316–1326. https://doi.org/10.1210/clinem/dgaa096.
53. Barrier, B.F.; Dick, E.J., Jr.; Butler, S.D.; Hubbard, G.B. Endometriosis involving the ileocaecal junction with regional lymph node involvement in the baboon--striking pathological finding identical between the human and the baboon: A case report. *Hum. Reprod.* **2007**, *22*, 272–274. https://doi.org/10.1093/humrep/del352.
54. Kim, J.J.; Taylor, H.S.; Lu, Z.; Ladhani, O.; Hastings, J.M.; Jackson, K.S.; Wu, Y.; Guo, S.W.; Fazleabas, A.T. Altered expression of HOXA10 in endometriosis: Potential role in decidualization. *Mol. Hum. Reprod.* **2007**, *13*, 323–332. https://doi.org/10.1093/molehr/gam005.
55. Lebovic, D.I.; Mwenda, J.M.; Chai, D.C.; Mueller, M.D.; Santi, A.; Fisseha, S.; D'Hooghe, T. PPAR-gamma receptor ligand induces regression of endometrial explants in baboons: A prospective, randomized, placebo- and drug-controlled study. *Fertil. Steril.* **2007**, *88*, 1108–1119. https://doi.org/10.1016/j.fertnstert.2006.12.072.
56. Falconer, H.; Mwenda, J.M.; Chai, D.C.; Song, X.Y.; Cornillie, F.J.; Bergqvist, A.; Fried, G.; D'Hooghe, T.M. Effects of anti-TNF-mAb treatment on pregnancy in baboons with induced endometriosis. *Fertil. Steril.* **2008**, *89*, 1537–1545. https://doi.org/10.1016/j.fertnstert.2007.05.062.
57. Bennett, M.W.; Dick, E.J., Jr.; Schlabritz-Loutsevitch, N.E.; Lopez-Alvarenga, J.C.; Williams, P.C.; Sharp, R.M.; Hubbard, G.B. Endometrial and cervical polyps in 22 baboons (Papio sp.), 5 cynomolgus macaques (*Macaca fascicularis*) and one marmoset (*Callithrix jacchus*). *J. Med. Primatol.* **2009**, *38*, 257–262. https://doi.org/10.1111/j.1600-0684.2009.00350.x.
58. Kyama, C.M.; Mihalyi, A.; Simsa, P.; Falconer, H.; Fulop, V.; Mwenda, J.M.; Peeraer, K.; Tomassetti, C.; Meuleman, C.; D'Hooghe, T.M. Role of cytokines in the endometrial-peritoneal cross-talk and development of endometriosis. *Front Biosci.* **2009**, *1*, 444–454. https://doi.org/10.2741/e40.
59. Jones, C.J.; Nardo, L.G.; Litta, P.; Fazleabas, A.T. Ultrastructure of ectopic peritoneal lesions from women with endometriosis, including observations on the contribution of coelomic mesothelium. *Reprod. Sci.* **2009**, *16*, 43–55. https://doi.org/10.1177/1933719108324891.
60. Winterhager, E.; Grümmer, R.; Mavrogianis, P.A.; Jones, C.J.; Hastings, J.M.; Fazleabas, A.T. Connexin expression pattern in the endometrium of baboons is influenced by hormonal changes and the presence of endometriotic lesions. *Mol. Hum. Reprod.* **2009**, *15*, 645–652. https://doi.org/10.1093/molehr/gap060.
61. Banerjee, P.; Fazleabas, A.T. Endometrial responses to embryonic signals in the primate. *Int. J. Dev. Biol.* **2010**, *54*, 295–302. https://doi.org/10.1387/ijdb.082829pb.
62. Fazleabas, A.T. Progesterone resistance in a baboon model of endometriosis. *Semin. Reprod. Med.* **2010**, *28*, 75–80. https://doi.org/10.1055/s-0029-1242997.
63. Joshi, N.R.; Miyadahira, E.H.; Afshar, Y.; Jeong, J.W.; Young, S.L.; Lessey, B.A.; Serafini, P.C.; Fazleabas, A.T. Progesterone Resistance in Endometriosis Is Modulated by the Altered Expression of MicroRNA-29c and FKBP4. *J. Clin. Endocrinol. Metab.* **2017**, *102*, 141–149. https://doi.org/10.1210/jc.2016-2076.

64. Sherwin, J.R.; Hastings, J.M.; Jackson, K.S.; Mavrogianis, P.A.; Sharkey, A.M.; Fazleabas, A.T. The endometrial response to chorionic gonadotropin is blunted in a baboon model of endometriosis. *Endocrinology* **2010**, *151*, 4982–4993. https://doi.org/10.1210/en.2010-0275.
65. Hapangama, D.K.; Turner, M.A.; Drury, J.; Heathcote, L.; Afshar, Y.; Mavrogianis, P.A.; Fazleabas, A.T. Aberrant expression of regulators of cell-fate found in eutopic endometrium is found in matched ectopic endometrium among women and in a baboon model of endometriosis. *Hum. Reprod.* **2010**, *25*, 2840–2850. https://doi.org/10.1093/humrep/deq248.
66. Ilad, R.S.; Fleming, S.D.; Murphy, C.R.; Fazleabas, A.T. Immunohistochemical study of the ubiquitin-nuclear factor-kB pathway in the endometrium of the baboon (*Papio anubis*) with and without endometriosis. *Reprod. Fertil. Dev.* **2010**, *22*, 1118–1130. https://doi.org/10.1071/rd08086.
67. Braundmeier, A.G.; Fazleabas, A.T.; Nowak, R.A. Extracellular matrix metalloproteinase inducer expression in the baboon endometrium: Menstrual cycle and endometriosis. *Reproduction* **2010**, *140*, 911–920. https://doi.org/10.1530/rep-09-0481.
68. Brosens, J.J.; Hodgetts, A.; Feroze-Zaidi, F.; Sherwin, J.R.; Fusi, L.; Salker, M.S.; Higham, J.; Rose, G.L.; Kajihara, T.; Young, S.L.; et al. Proteomic analysis of endometrium from fertile and infertile patients suggests a role for apolipoprotein A-I in embryo implantation failure and endometriosis. *Mol. Hum. Reprod.* **2010**, *16*, 273–285. https://doi.org/10.1093/molehr/gap108.
69. Lebovic, D.I.; Mwenda, J.M.; Chai, D.C.; Santi, A.; Xu, X.; D'Hooghe, T. Peroxisome proliferator-activated receptor-(gamma) receptor ligand partially prevents the development of endometrial explants in baboons: A prospective, randomized, placebo-controlled study. *Endocrinology* **2010**, *151*, 1846–1852. https://doi.org/10.1210/en.2009-1076.
70. Hey-Cunningham, A.J.; Fazleabas, A.T.; Braundmeier, A.G.; Markham, R.; Fraser, I.S.; Berbic, M. Endometrial stromal cells and immune cell populations within lymph nodes in a nonhuman primate model of endometriosis. *Reprod. Sci.* **2011**, *18*, 747–754. https://doi.org/10.1177/1933719110397210.
71. Morris, K.; Ihnatovych, I.; Ionetz, E.; Reed, J.; Braundmeier, A.; Strakova, Z. Cofilin and slingshot localization in the epithelium of uterine endometrium changes during the menstrual cycle and in endometriosis. *Reprod. Sci.* **2011**, *18*, 1014–1024. https://doi.org/10.1177/1933719111401663.
72. Kemnitz, J.W. Calorie restriction and aging in nonhuman primates. *Ilar J.* **2011**, *52*, 66–77. https://doi.org/10.1093/ilar.52.1.66.
73. Harirchian, P.; Gashaw, I.; Lipskind, S.T.; Braundmeier, A.G.; Hastings, J.M.; Olson, M.R.; Fazleabas, A.T. Lesion kinetics in a non-human primate model of endometriosis. *Hum. Reprod.* **2012**, *27*, 2341–2351. https://doi.org/10.1093/humrep/des196.
74. Campo, S.; Campo, V.; Benagiano, G. Infertility and adenomyosis. *Obstet. Gynecol. Int.* **2012**, *2012*, 786132. https://doi.org/10.1155/2012/786132.
75. Afshar, Y.; Hastings, J.; Roqueiro, D.; Jeong, J.W.; Giudice, L.C.; Fazleabas, A.T. Changes in eutopic endometrial gene expression during the progression of experimental endometriosis in the baboon, *Papio anubis*. *Biol. Reprod.* **2013**, *88*, 44. https://doi.org/10.1095/biolreprod.112.104497.
76. Langoi, D.; Pavone, M.E.; Gurates, B.; Chai, D.; Fazleabas, A.; Bulun, S.E. Aromatase inhibitor treatment limits progression of peritoneal endometriosis in baboons. *Fertil. Steril.* **2013**, *99*, 656–662.e653. https://doi.org/10.1016/j.fertnstert.2012.11.021.
77. Jagirdar, J.; Sirohi, D.; Dick, E.J., Jr.; Hubbard, G. Pleuro-pulmonary endometriosis in baboons (*Papio* spp.): Insights into pathogenesis. *J. Med. Primatol.* **2013**, *42*, 39–45. https://doi.org/10.1111/jmp.12027.
78. Donnez, O.; Van Langendonckt, A.; Defrère, S.; Colette, S.; Van Kerk, O.; Dehoux, J.P.; Squifflet, J.; Donnez, J. Induction of endometriotic nodules in an experimental baboon model mimicking human deep nodular lesions. *Fertil. Steril.* **2013**, *99*, 783–789.e3. https://doi.org/10.1016/j.fertnstert.2012.10.032.
79. Donnez, O.; Soares, M.; Defrère, S.; Dehoux, J.P.; van Langendonckt, A.; Donnez, J.; Dolmans, M.M.; Colette, S. Nerve fiber density in deep nodular endometriotic lesions induced in a baboon experimental model. *Fertil. Steril.* **2013**, *100*, 1144–1150. https://doi.org/10.1016/j.fertnstert.2013.06.014.
80. Orellana, R.; García-Solares, J.; Donnez, J.; van Kerk, O.; Dolmans, M.M.; Donnez, O. Important role of collective cell migration and nerve fiber density in the development of deep nodular endometriosis. *Fertil. Steril.* **2017**, *107*, 987–995.e5. https://doi.org/10.1016/j.fertnstert.2017.01.005.
81. Kyama, C.M.; Falconer, H.; Cuneo, S.; Chai, D.; Mihalyi, A.; Mwenda, J.; D'Hooghe, T. Menstrual endometrial supernatant may induce stromal endometriosis in baboons. *Front Biosci.* **2014**, *6*, 16–28. https://doi.org/10.2741/s410.

82. Sugihara, K.; Kobayashi, Y.; Suzuki, A.; Tamura, N.; Motamedchaboki, K.; Huang, C.T.; Akama, T.O.; Pecotte, J.; Frost, P.; Bauer, C.; et al. Development of pro-apoptotic peptides as potential therapy for peritoneal endometriosis. *Nat. Commun.* **2014**, *5*, 4478. https://doi.org/10.1038/ncomms5478.
83. Fazleabas, A.T.; Braundmeier, A.; Parkin, K. Endometriosis-induced changes in regulatory T cells—Insights towards developing permanent contraception. *Contraception* **2015**, *92*, 116–119. https://doi.org/10.1016/j.contraception.2015.06.006.
84. Braundmeier, A.; Jackson, K.; Hastings, J.; Koehler, J.; Nowak, R.; Fazleabas, A. Induction of endometriosis alters the peripheral and endometrial regulatory T cell population in the non-human primate. *Hum. Reprod.* **2012**, *27*, 1712–1722. https://doi.org/10.1093/humrep/des083.
85. Joshi, N.R.; Su, R.W.; Chandramouli, G.V.; Khoo, S.K.; Jeong, J.W.; Young, S.L.; Lessey, B.A.; Fazleabas, A.T. Altered expression of microRNA-451 in eutopic endometrium of baboons (*Papio anubis*) with endometriosis. *Hum. Reprod.* **2015**, *30*, 2881–2891. https://doi.org/10.1093/humrep/dev229.
86. Nothnick, W.B.; Falcone, T.; Joshi, N.; Fazleabas, A.T.; Graham, A. Serum miR-451a Levels Are Significantly Elevated in Women with Endometriosis and Recapitulated in Baboons (*Papio anubis*) with Experimentally-Induced Disease. *Reprod. Sci.* **2017**, *24*, 1195–1202. https://doi.org/10.1177/1933719116681519.
87. Kim, B.G.; Yoo, J.Y.; Kim, T.H.; Shin, J.H.; Langenheim, J.F.; Ferguson, S.D.; Fazleabas, A.T.; Young, S.L.; Lessey, B.A.; Jeong, J.W. Aberrant activation of signal transducer and activator of transcription-3 (STAT3) signaling in endometriosis. *Hum. Reprod.* **2015**, *30*, 1069–1078. https://doi.org/10.1093/humrep/dev050.
88. Yoo, J.Y.; Jeong, J.W.; Fazleabas, A.T.; Tayade, C.; Young, S.L.; Lessey, B.A. Protein Inhibitor of Activated STAT3 (PIAS3) Is Down-Regulated in Eutopic Endometrium of Women with Endometriosis. *Biol. Reprod.* **2016**, *95*, 11. https://doi.org/10.1095/biolreprod.115.137158.
89. Su, R.W.; Fazleabas, A.T. Implantation and Establishment of Pregnancy in Human and Nonhuman Primates. *Adv. Anat. Embryol. Cell Biol.* **2015**, *216*, 189–213. https://doi.org/10.1007/978-3-319-15856-3_10.
90. Baumann, C.; Olson, M.; Wang, K.; Fazleabas, A.; De La Fuente, R. Arginine methyltransferases mediate an epigenetic ovarian response to endometriosis. *Reproduction* **2015**, *150*, 297–310. https://doi.org/10.1530/rep-15-0212.
91. Zhang, Q.; Duan, J.; Olson, M.; Fazleabas, A.; Guo, S.W. Cellular Changes Consistent with Epithelial-Mesenchymal Transition and Fibroblast-to-Myofibroblast Transdifferentiation in the Progression of Experimental Endometriosis in Baboons. *Reprod. Sci.* **2016**, *23*, 1409–1421. https://doi.org/10.1177/1933719116641763.
92. Parkin, K.L.; Fazleabas, A.T. Uterine Leukocyte Function and Dysfunction: A Hypothesis on the Impact of Endometriosis. *Am. J. Reprod. Immunol.* **2016**, *75*, 411–417. https://doi.org/10.1111/aji.12487.
93. Slayden, O.D. Translational In Vivo Models for Women's Health: The Nonhuman Primate Endometrium--A Predictive Model for Assessing Steroid Receptor Modulators. *Handb. Exp. Pharmacol.* **2016**, *232*, 191–202. https://doi.org/10.1007/164_2015_22.
94. Hussein, M.; Chai, D.C.; Kyama, C.M.; Mwenda, J.M.; Palmer, S.S.; Gotteland, J.P.; D'Hooghe, T.M. c-Jun NH2-terminal kinase inhibitor bentamapimod reduces induced endometriosis in baboons: An assessor-blind placebo-controlled randomized study. *Fertil. Steril.* **2016**, *105*, 815–824.e5. https://doi.org/10.1016/j.fertnstert.2015.11.022.
95. Taylor, H.S.; Alderman Iii, M.; D'Hooghe, T.M.; Fazleabas, A.T.; Duleba, A.J. Effect of simvastatin on baboon endometriosis. *Biol. Reprod.* **2017**, *97*, 32–38. https://doi.org/10.1093/biolre/iox058.
96. Cosar, E.; Mamillapalli, R.; Moridi, I.; Duleba, A.; Taylor, H.S. Serum MicroRNA Biomarkers Regulated by Simvastatin in a Primate Model of Endometriosis. *Reprod. Sci.* **2019**, *26*, 1343–1350. https://doi.org/10.1177/1933719118765971.
97. Yoo, J.Y.; Kim, T.H.; Fazleabas, A.T.; Palomino, W.A.; Ahn, S.H.; Tayade, C.; Schammel, D.P.; Young, S.L.; Jeong, J.W.; Lessey, B.A. KRAS Activation and over-expression of SIRT1/BCL6 Contributes to the Pathogenesis of Endometriosis and Progesterone Resistance. *Sci. Rep.* **2017**, *7*, 6765. https://doi.org/10.1038/s41598-017-04577-w.
98. Stouffer, R.L.; Woodruff, T.K. Nonhuman Primates: A Vital Model for Basic and Applied Research on Female Reproduction, Prenatal Development, and Women's Health. *Ilar J.* **2017**, *58*, 281–294. https://doi.org/10.1093/ilar/ilx027.
99. Drury, J.A.; Parkin, K.L.; Coyne, L.; Giuliani, E.; Fazleabas, A.T.; Hapangama, D.K. The dynamic changes in the number of uterine natural killer cells are specific to the eutopic but not to the ectopic endometrium in women and in a baboon model of endometriosis. *Reprod. Biol. Endocrinol.* **2018**, *16*, 67. https://doi.org/10.1186/s12958-018-0385-3.
100. Chang, H.J.; Yoo, J.Y.; Kim, T.H.; Fazleabas, A.T.; Young, S.L.; Lessey, B.A.; Jeong, J.W. Overexpression of Four Joint Box-1 Protein (FJX1) in Eutopic Endometrium From Women with Endometriosis. *Reprod. Sci.* **2018**, *25*, 207–213. https://doi.org/10.1177/1933719117716780.

101. Hufnagel, D.; Goetz, T.G.; Hu, Z.; Nyachieo, A.; D'Hooghe, T.; Fazleabas, A.; Duleba, A.; Krikun, G.; Taylor, H.S.; Lockwood, C.J. Icon immunoconjugate treatment results in regression of red lesions in a non-human primate (*Papio anubis*) model of endometriosis. *Reprod. Biol.* **2018**, *18*, 109–114. https://doi.org/10.1016/j.repbio.2018.01.009.
102. Nothnick, W.B.; Falcone, T.; Olson, M.R.; Fazleabas, A.T.; Tawfik, O.W.; Graham, A. Macrophage Migration Inhibitory Factor Receptor, CD74, is Overexpressed in Human and Baboon (*Papio anubis*) Endometriotic Lesions and Modulates Endometriotic Epithelial Cell Survival and Interleukin 8 Expression. *Reprod. Sci.* **2018**, *25*, 1557–1566. https://doi.org/10.1177/1933719118766262.
103. Kim, T.H.; Yoo, J.Y.; Choi, K.C.; Shin, J.H.; Leach, R.E.; Fazleabas, A.T.; Young, S.L.; Lessey, B.A.; Yoon, H.G.; Jeong, J.W. Loss of HDAC3 results in nonreceptive endometrium and female infertility. *Sci. Transl. Med.* **2019**, *11*, eaaf7533. https://doi.org/10.1126/scitranslmed.aaf7533.
104. Hapangama, D.K.; Drury, J.; Da Silva, L.; Al-Lamee, H.; Earp, A.; Valentijn, A.J.; Edirisinghe, D.P.; Murray, P.A.; Fazleabas, A.T.; Gargett, C.E. Abnormally located SSEA1+/SOX9+ endometrial epithelial cells with a basalis-like phenotype in the eutopic functionalis layer may play a role in the pathogenesis of endometriosis. *Hum. Reprod.* **2019**, *34*, 56–68. https://doi.org/10.1093/humrep/dey336.
105. Kirejczyk, S.; Pinelli, C.; Gonzalez, O.; Kumar, S.; Dick, E., Jr.; Gumber, S. Urogenital Lesions in Nonhuman Primates at 2 National Primate Research Centers. *Vet. Pathol.* **2021**, *58*, 147–160. https://doi.org/10.1177/0300985820971752.
106. Le, N.; Cregger, M.; Fazleabas, A.; Braundmeier-Fleming, A. Effects of endometriosis on immunity and mucosal microbial community dynamics in female olive baboons. *Sci. Rep.* **2022**, *12*, 1590. https://doi.org/10.1038/s41598-022-05499-y.
107. Poirier, D.; Nyachieo, A.; Romano, A.; Roy, J.; Maltais, R.; Chai, D.; Delvoux, B.; Tomassetti, C.; Vanhie, A. An irreversible inhibitor of 17β-hydroxysteroid dehydrogenase type 1 inhibits estradiol synthesis in human endometriosis lesions and induces regression of the non-human primate endometriosis. *J. Steroid Biochem. Mol. Biol.* **2022**, *222*, 106136. https://doi.org/10.1016/j.jsbmb.2022.106136.
